# Melioidosis Presenting as a Solitary Liver Abscess With Culture-Negative Septic Arthritis: A Case Report

**DOI:** 10.7759/cureus.116326

**Published:** 2026-09-15

**Authors:** Zubin Vaid, Ankit B Shah, Dhruti Sheth, Ami Varaiya

**Affiliations:** 1 Medicine, Dr. Balabhai Nanavati Hospital, Mumbai, IND; 2 Radiology, Eclat Imaging Centre, Mumbai, IND; 3 Microbiology, Agilus Diagnostics Dr. Phadke Labs, Mumbai, IND; 4 Microbiology, Nanavati Max Super Speciality Hospital, Mumbai, IND

**Keywords:** burkholderia pseudomallei, diabetes mellitus, melioidosis, septic arthritis, solitary liver abscess

## Abstract

Melioidosis is an infection caused by *Burkholderia pseudomallei* with varied clinical manifestations, particularly in patients with diabetes mellitus. Hepatic involvement usually occurs as part of multifocal disease, while a solitary liver abscess is uncommon. We report a 49-year-old man with poorly controlled type 2 diabetes mellitus who presented with fever and right upper quadrant pain. Ultrasonography showed a right lobe liver abscess, and empirical treatment with ceftriaxone and metronidazole was started. Despite treatment, he developed persistent fever, increasing leukocytosis, hypotension, metabolic acidosis, altered mentation, and acute painful swelling of the left knee. Contrast-enhanced computed tomography showed a solitary multiloculated liver abscess measuring 5.3 × 3.8 × 3.2 cm with a honeycomb appearance. Percutaneous drainage was performed, and culture of the liver abscess grew *Burkholderia pseudomallei*. Ultrasound-guided aspiration of the knee yielded purulent hemorrhagic fluid with a leukocyte count of 35,000/mm³ and low glucose. Gram stain and culture of the joint fluid were negative, and the findings were considered consistent with culture-negative septic arthritis associated with melioidosis. The patient was treated with intravenous ceftazidime with marked clinical improvement within 36-48 hours, followed by oral trimethoprim-sulfamethoxazole eradication therapy. He remained clinically well at approximately one-year follow-up. This case highlights that melioidosis should be considered in patients with diabetes with a solitary liver abscess, particularly when there is poor response to empirical therapy and evidence of another infective focus.

## Introduction

In India, amoebic and pyogenic abscesses account for most cases of liver abscess [1,2]. *Escherichia coli* and *Klebsiella pneumoniae* are important Gram-negative causes of pyogenic liver abscess [1,2]. Melioidosis, caused by *Burkholderia pseudomallei*, is increasingly recognized in India but remains underdiagnosed because of its variable clinical presentation [3,4]. Visceral abscesses are a well-recognized manifestation of melioidosis [5]. Hepatic involvement usually occurs as part of multifocal or disseminated disease, while presentation as a solitary liver abscess is uncommon [5,6]. The wide clinical spectrum of melioidosis can mimic other infections and delay appropriate diagnosis and treatment [3,7]. We report a case of culture-confirmed melioidosis presenting as a solitary liver abscess with associated culture-negative septic arthritis in a patient with poorly controlled diabetes mellitus.

## Case presentation

A 49-year-old man with long-standing poorly controlled type 2 diabetes mellitus presented with fever for two weeks associated with moderate right upper quadrant abdominal pain. He worked as a supervisor at construction sites but reported no direct occupational exposure to soil, surface water, or muddy environments. There was no history of diarrheal illness. Mild respiratory distress was noted at presentation. Laboratory investigations showed leukocytosis, hyperglycemia, poor glycemic control, and elevated liver transaminases, as summarized in Table 1.

**Table 1:** Laboratory investigations at presentation HbA1c: glycated hemoglobin, AST: aspartate aminotransferase, ALT: alanine aminotransferase

Parameter	Patient value	Reference range
Total leukocyte count (cells/mm³)	15,300	4,000-11,000
Random blood glucose (mg/dL)	240	70-140
HbA1c (%)	9.8	<5.7
AST (U/L)	240	5-40
ALT (U/L)	310	7-56

Abdominal ultrasonography showed a liver abscess in the right lobe (Figure 1). A provisional diagnosis of amoebic liver abscess was considered, with pyogenic liver abscess as a differential diagnosis. The patient was started empirically on intravenous ceftriaxone and metronidazole. Blood cultures obtained after initiation of empirical antimicrobial therapy remained sterile.

**Figure 1:**
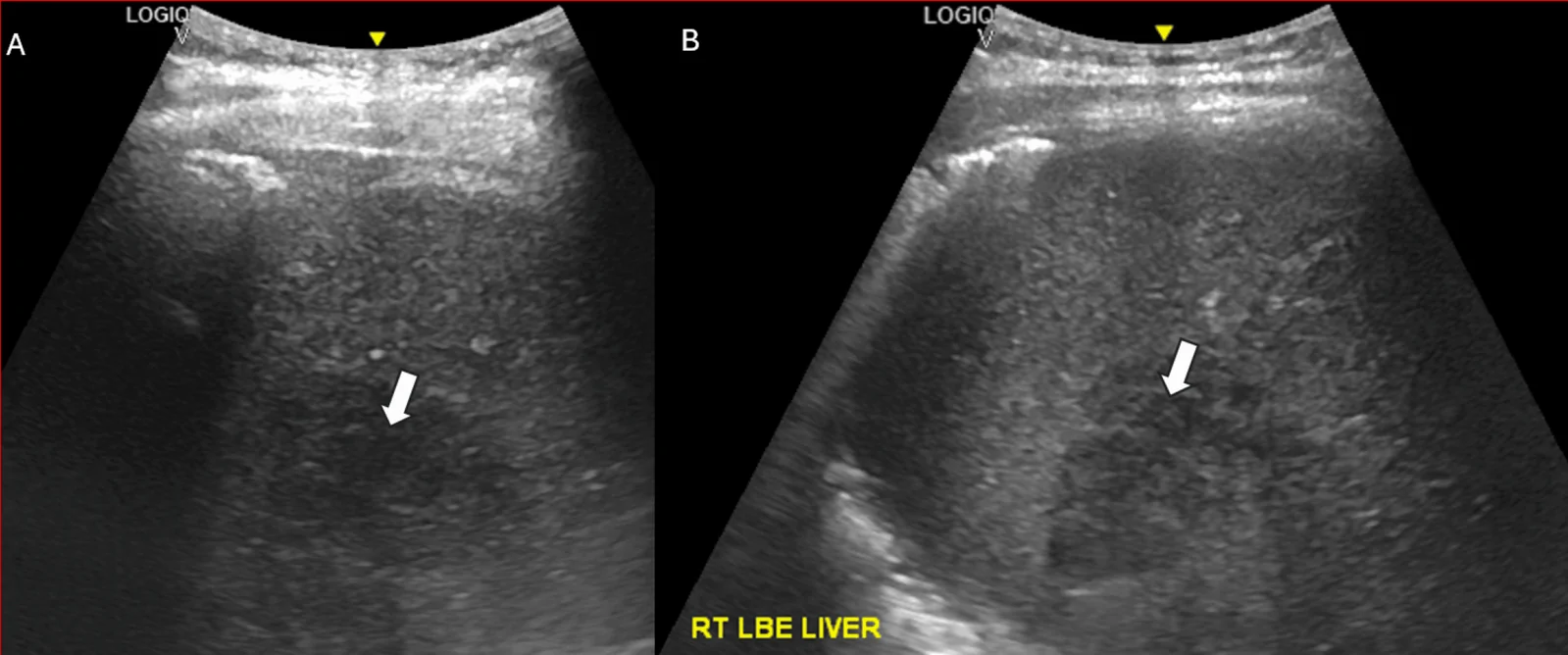
Abdominal ultrasonography showing a right lobe liver abscess Ultrasonography of the liver showing a right lobe abscess. (A) Short-axis and (B) longitudinal grayscale images demonstrate a poorly marginated, partially liquefied complex cystic lesion in segment VII of the right hepatic lobe (arrows).

There was no clinical improvement over the next 48 hours. The patient continued to have high-grade fever, and the total leukocyte count increased to 20,080/mm³. His condition progressively deteriorated with hypotension, high anion gap metabolic acidosis, altered mentation, and increasing abdominal tenderness with guarding.

During this period, he developed acute pain and swelling of the left knee. There was no previous history of arthritis or recurrent joint symptoms. Clinical examination revealed a tender effusion, and ultrasonography of the left knee confirmed moderate joint effusion (Figure 2).

**Figure 2:**
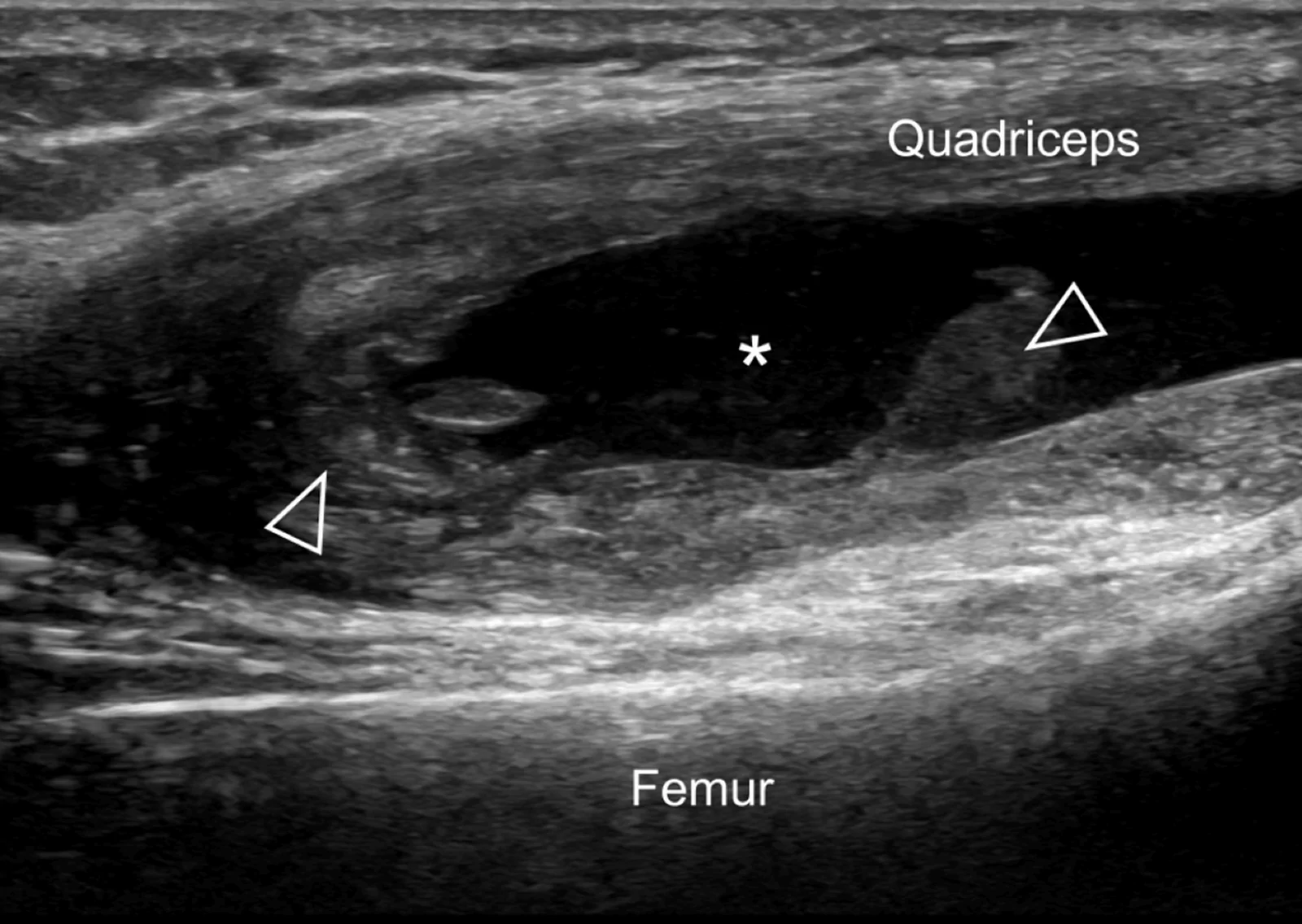
Ultrasonography of the left knee showing joint effusion Ultrasonography of the left knee showing joint effusion. The long-axis image of the suprapatellar recess demonstrates moderate joint effusion (asterisk) with associated synovial hypertrophy (open triangles).

Contrast-enhanced computed tomography of the abdomen was subsequently performed. It showed a solitary multiloculated liver abscess measuring 5.3 × 3.8 × 3.2 cm with a honeycomb appearance (Figure 3). No other hepatic abscess was identified. Percutaneous drainage of the liver abscess using a pigtail catheter was performed, and frank pus was obtained for microbiological examination.

**Figure 3:**
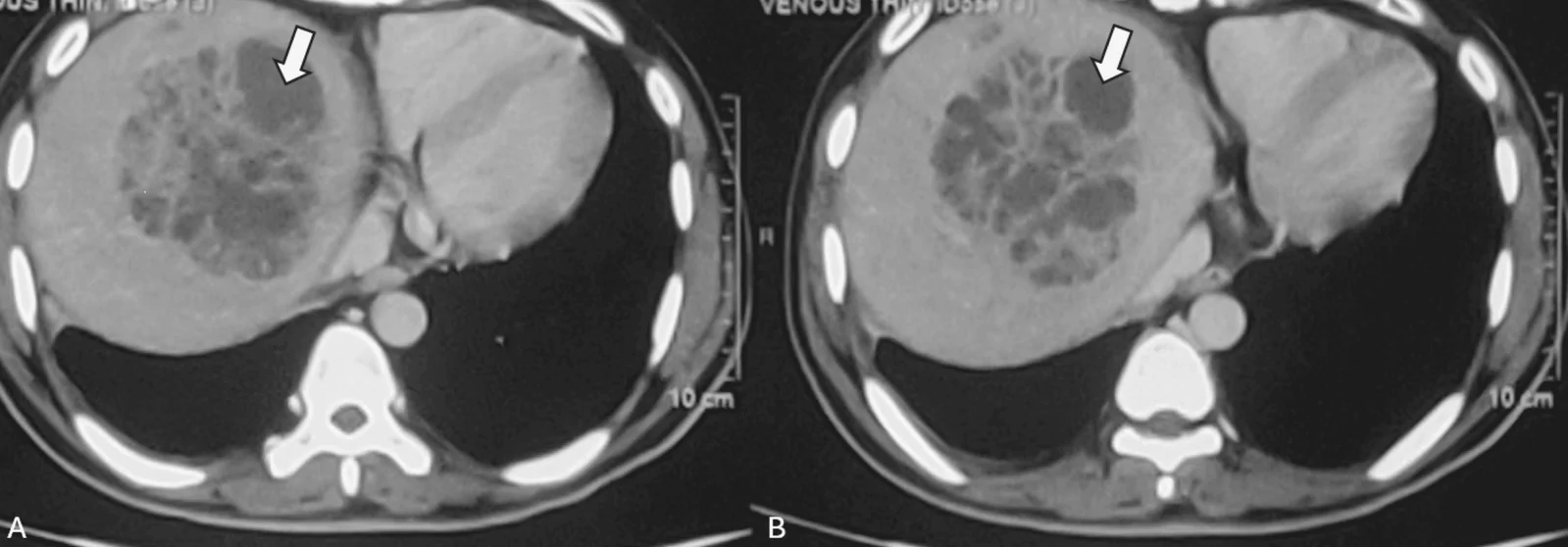
Contrast-enhanced computed tomography of the abdomen showing a solitary multiloculated liver abscess with a honeycomb appearance Contrast-enhanced computed tomography of the abdomen showing a multiloculated abscess in the right hepatic lobe. (A and B) Axial post-contrast images demonstrate peripheral enhancement with multiple central non-enhancing locules (arrows), producing a honeycomb appearance. The lesion measured 5.3 × 3.8 × 3.2 cm.

Gram staining of the liver abscess aspirate showed abundant pus cells without demonstrable organisms. Ziehl-Neelsen staining did not show acid-fast bacilli, GeneXpert for *Mycobacterium tuberculosis* was negative, and potassium hydroxide mount did not reveal fungal elements. After 48 hours of incubation, culture of the pus grew Gram-negative bacilli on blood agar, chocolate agar, and MacConkey agar. Gram staining of the culture growth showed Gram-negative bacilli with bipolar staining. MacConkey agar showed non-lactose-fermenting wrinkled colonies with a metallic sheen.

Identification and antimicrobial susceptibility testing were performed using the VITEK 2 Compact system (bioMérieux, Marcy-l’Étoile, France). The isolate was identified as *Burkholderia pseudomallei* with 99% probability. It was susceptible to ceftazidime, imipenem, meropenem, amoxicillin-clavulanic acid, trimethoprim-sulfamethoxazole, and doxycycline.

Ultrasound-guided aspiration of the left knee was performed once, and approximately 7-8 mL of thick purulent hemorrhagic fluid was aspirated. Synovial fluid analysis showed a leukocyte count of 35,000/mm³ with 92% neutrophils and low glucose. No crystals were identified. Gram stain did not show any organism, and the bacterial culture remained sterile. No radiograph or magnetic resonance imaging of the knee was performed. The patient did not undergo arthroscopic or open joint washout.

In view of the purulent joint aspirate, synovial leukocytosis, low synovial glucose, and concurrent culture-confirmed melioidosis, the knee involvement was considered consistent with culture-negative septic arthritis associated with melioidosis. Since *B. pseudomallei* was not isolated from the synovial fluid, direct microbiological confirmation of melioidotic arthritis was not available.

The patient was started on intravenous ceftazidime 2 g every eight hours after identification of *B. pseudomallei*. A marked clinical response was observed within 36-48 hours. Fever and features of sepsis improved, along with significant improvement in left knee pain and swelling. Intravenous ceftazidime was continued for two weeks, followed by oral trimethoprim-sulfamethoxazole for 12 weeks as eradication therapy.

The patient remained clinically well after completion of treatment. He was last followed up in July 2025, approximately one year after the initial illness, and remained asymptomatic without clinically evident recurrence.

## Discussion

Melioidosis is increasingly recognized in India but remains underdiagnosed because of its varied clinical presentation [3,4]. Reliable population-based incidence data are limited. A modeling study estimated approximately 52,000 cases and 31,425 deaths annually in India, suggesting a considerably higher burden than that reflected by reported cases [4,8]. Diabetes mellitus is the most important predisposing factor. Recent Indian studies have reported diabetes in approximately 73%-75% of patients, while alcohol use, chronic kidney disease, chronic lung disease, malignancy, and immunosuppression are other recognized risk factors [4,9]. Our patient had poorly controlled diabetes with an HbA1c of 9.8%, which was the major identified predisposing factor. He worked as a supervisor at construction sites but reported no direct occupational exposure to soil, surface water, or muddy environments.

Melioidosis can involve several organ systems. Clinical presentations include pneumonia, skin and soft tissue infection, genitourinary disease, visceral abscesses, neurological disease, and musculoskeletal involvement [7,9,10]. Musculoskeletal disease may present as osteomyelitis, septic arthritis, or soft tissue abscesses [10]. A recent systematic review of 558 confirmed cases from India reported bone or joint involvement in 35.1%, pulmonary disease in 33.2%, hepatic abscesses in 12.5%, and splenic abscesses in 17.7% [9]. These findings highlight the wide clinical spectrum of the disease and the potential for involvement of more than one anatomical site.

Visceral abscesses are well-recognized manifestations of melioidosis, with the liver and spleen being commonly involved [5]. Hepatic involvement often occurs with multiple or multiloculated lesions, while presentation as a solitary liver abscess is uncommon [5,6]. Multiloculated hepatic abscesses with small locules separated by internal septa may produce a honeycomb appearance on computed tomography [5,11]. This finding can provide an important radiological clue but is not specific for melioidosis. Microbiological confirmation remains essential [11]. In our patient, poorly controlled diabetes, failure to respond to initial ceftriaxone and metronidazole, progression to sepsis, development of a second infective focus in the knee, and the honeycomb appearance of the liver abscess raised suspicion of melioidosis. The diagnosis was subsequently confirmed by culture of the liver aspirate, which grew *Burkholderia pseudomallei*.

Blood cultures may remain negative in clinically significant melioidosis because bacteremia is not present in all patients. In a recent Indian systematic review, blood cultures were positive in 288 of 411 (70.1%) patients with available blood-culture data [9]. In our patient, blood cultures were obtained after initiation of empirical ceftriaxone and metronidazole and remained sterile, while *B. pseudomallei* was subsequently isolated from the liver abscess. Prior antimicrobial exposure may also have reduced the culture yield.

Osteoarticular melioidosis is an important but less frequent manifestation of the disease [12]. In the Darwin Prospective Melioidosis Study, septic arthritis was identified in 62 of 1,129 (5.5%) patients with culture-confirmed melioidosis. The lower limb was most frequently affected, with the knee being the most commonly involved joint. Diabetes was present in 69.6% of patients with osteoarticular disease [12]. In our patient, acute monoarthritis of the left knee developed during clinical deterioration, with no previous history of arthritis or recurrent joint symptoms. Aspiration yielded purulent hemorrhagic fluid with a leukocyte count of 35,000/mm³, 92% neutrophils, and low glucose. These findings supported septic arthritis. However, *B. pseudomallei* was not isolated from the synovial fluid, and hence, melioidotic arthritis could not be microbiologically confirmed. The temporal association with hepatic melioidosis and the rapid improvement in both systemic illness and knee symptoms after ceftazidime supported probable secondary joint involvement.

Source control is an important part of the management of melioidosis, particularly in patients with accessible abscesses and osteoarticular disease [12,13]. In our patient, percutaneous pigtail drainage of the hepatic abscess provided therapeutic source control as well as material for microbiological diagnosis. The knee was treated with a single ultrasound-guided aspiration. Surgical washout is commonly required in osteoarticular melioidosis, particularly when there is inadequate clinical response or persistent infection [12,13]. However, the knee pain and swelling in our patient improved markedly after aspiration and initiation of ceftazidime. Arthroscopic or open washout was therefore not performed.

Treatment of melioidosis consists of an intensive intravenous phase followed by prolonged oral eradication therapy. General recommendations describe an intensive phase of approximately two to four weeks, with longer treatment according to the site and severity of infection [7]. Current Australian guidance recommends approximately four weeks of intravenous therapy followed by three months of oral eradication treatment for confirmed septic arthritis [13]. Our patient showed a marked clinical response within 36-48 hours of starting ceftazidime, with resolution of sepsis and significant improvement in knee symptoms. As *B. pseudomallei* was not isolated from the synovial fluid and the joint involvement was not microbiologically confirmed, the treating team continued intravenous ceftazidime for 2 weeks followed by oral trimethoprim-sulfamethoxazole for 12 weeks. This intensive phase was shorter than the current site-specific recommendations for confirmed septic arthritis. However, the patient remained clinically well without recurrence at approximately one-year follow-up. Dedicated radiological evaluation of the knee was not performed to exclude associated osteomyelitis, which remains a limitation of this case.

## Conclusions

Melioidosis should be considered in patients with diabetes presenting with a solitary liver abscess, particularly when there is poor response to conventional empirical therapy. Characteristic imaging findings such as a honeycomb appearance and the development of a second infective focus may provide important diagnostic clues. In this case, culture-confirmed hepatic melioidosis was associated with culture-negative septic arthritis, with rapid clinical improvement after initiation of ceftazidime. Early microbiological diagnosis and appropriate antimicrobial therapy remain important in preventing the progression of this potentially severe infection.
